# Nutritional profiling of some selected commercially important freshwater and marine water fishes of Bangladesh

**DOI:** 10.1016/j.heliyon.2022.e10825

**Published:** 2022-09-29

**Authors:** Md. Rahamat Ullah, Md. Arifur Rahman, Md. Nazmul Haque, Md. Rajib Sharker, M. Muhsinul Islam, Md. Ariful Alam

**Affiliations:** a Bangladesh Fisheries Research Institute, Riverine Sub-Station, Khepupara, Patuakhali, 8650, Bangladesh; b Department of Fisheries Biology and Genetics, Patuakhali Science and Technology University, Dumki, Patuakhali, 8602, Bangladesh

**Keywords:** Proximate composition, Mineral content, Nutritive value, Freshwater, Marine water

## Abstract

The purpose of the study was to investigate the proximate composition and mineral content of Bangladesh's economically important freshwater and marine water fish. Proximate composition and mineral content was determined according to the Association of Official Agricultural Chemists (AOAC) standard method. All of the factors had a substantial variation (*p* < 0.05), according to the findings. The maximum protein content was observed in *Lates calcarifer* (18.673%) and minimum in *Pangasius pangasius* (15.616%). The content of lipid among the species varied from 0.316% to 13.396%, with *Mugil cephalus* having the highest lipid content and *Channa striata* having the lowest. The moisture content ranges from 68.343% to 81.160%. All the fishes have an average ash content of 0.850%–4.350%. The energy content is also significantly higher in marine water fishes. The mineral content was highly variable. Calcium content was lowest in *Pangasius pangasius* (0.555%) and highest in *Setipinna phasa* (3.495%). The magnesium content ranged between 0.281% and 1.885%. Phosphorus was lowest in *Lepturacanthus savala* (0.826%) and highest in *Setipinna phasa* (2.114%). The amount of sodium, potassium, and sulfur was relatively less for all fish species but there were substantial differences across the twelve samples. The PCA biplot's for proximate composition analyses has demonstrated positive affinity only between *Lates calcarifer* and *Mugil cephalus* in case of ash, lipid, and carbohydrate whereas *Setipina phasa*, *Mugil cephalus*, *Lutjanus lutjanus*, and *Oreochromis mossambicus* were grouped together with magnesium, phosphorus, calcium, and sulfur in the case of mineral content. Overall, the marine water fishes can be a good food item in terms of nutrition which could provide better health benefits for human.

## 1 Introduction

Fish is one of the most important animal protein sources accounting for 16% of entire animal protein consumed by the world's population and it's said to be an excellent provider of important nutrients for maintaining a healthy body (FAO, 2000; Ondo-Azi et al., 2013). Fish proteins have great biological significance as they contain enough amounts of necessary amino acids (Moujahed, 2015). According to numerous studies, the major source of EPA/DHA in the human diet has been obtained from natural marine and freshwater environments fish (Panchal and Brown, 2021). Fish consumption has been related to health advantages due to the long-chain PUFA's role in preventing coronary artery disease in humans, improving the development of the retina and brain, lowering the risk of breast cancer, asthma, inflammatory bowel disease, rheumatoid arthritis, and regulating prostaglandin synthesis (Raatz et al., 2013; Islam et al., 2021; Ullah et al., 2022).

Fish is rich in essential nutrients that can be utilized to enhance meals for both babies and childrens. Fish, on the other hand, is mostly made up of protein, fat, ash, and water, with minor quantities of polysaccharides and non-protein substances. The protein composition of most fish is between 15 and 30% of weight, the fat level is between 0 and 25%, and the moisture content is around 50–80% of body weight (Menon et al., 2010; Hasan et al., 2015; Hossain et al., 2015; Chakma et al., 2020). As opposed to this, minerals can play a pivotal role in maintaining acid-base and water balance, formation teeth structure and bones, and accelerating metabolic reactions in the human body (Zhang et al., 2020). In addition to their nutritional and physiological impacts, minerals are important as food additives because they enhance food flavor, promote or inhibit enzyme-catalyzed and other biochemical activities, and alter food texture (Ersoy and Celik, 2010). The muscle and bones of fish are rich in different essential nutrients like protein, amino acids, and fatty acids (Ersoy and Özeren, 2009; Zula and Desta, 2021).

However, within and across various water bodies and species the percentage of components may vary. As a result, it's critical to examine the biochemical makeup of protein, fat, ash, and mineral content of fish that we regularly consume. However, a comparative investigation of the proximate composition and mineral content of freshwater and marine water fish species in Bangladesh has turned up very little information. Hence, the current study was conducted to determine the proximate composition and mineral content of six economically significant freshwater and six marine water fish species that are highly essential in the cuisine of Bangladeshi people of the coastal region as a healthy food source.

## 2 Materials and methods

### 2.1 Sample collection

Six commercially significant freshwater fish species were collected from the Payra river in the Patuakhali district. However, six marine water fish samples were obtained from Mohipur Fish Landing Center in the Patuakhali district. The description of the fishes is provided in Table 1. The samples were transported to the Molecular Biology and Conservation Laboratory at Patuakhali Science and Technology University and then the Agri Chemistry Laboratory for subsequent examination in an insulated icebox with a fish-to-ice ratio of 1:2 (w/w). The research was conducted in accordance with the ethical standards of the Faculty of Fisheries at Patuakhali Science and Technology University. The experimental protocol and guidelines were maintained according to the Animal Welfare and Ethical Committee of Patuakhali Science and Technology University, Patuakhali, Bangladesh. The research and use of animals for the experiment have been authorized by the Ethical Committee.

**Table 1:** An overview of six freshwater and six marine water fish species collected and their habitat from Bangladesh.

Categories	Common Name	Scientific Name	Local Name (Bangladesh)	Tissue Sampled	Habitat
Freshwater fish	Indian major carp	*Labeo rohita*	Rui	Fillet	Pelagic
	Mozambique tilapia	*Oreochromis mossambicus*	Tilapia	Fillet	Benthopelagic
	Pangas catfish	*Pangasius pangasius*	Pangas	Fillet	Demersal
	Walking catfish	*Clarias batrachus*	Magur	Fillet with skin	Demersal
	Paradise threadfin	*Polynemus paradiseus*	Tapasi, Topse	Whole fish	Demersal
	Chevron snakehead	*Channa striata*	Shol	Fillet with skin	Benthopelagic
Marine water fish	Savalai hairtail	*Lepturacanthus savala*	Churi	Whole fish	Benthopelagic
	Asian seabass	*Lates calcarifer*	Bhetki, Koral	Fillet	Demersal
	Flathead grey mullet	*Mugil cephalus*	Mullet	Whole fish	Benthopelagic
	Gangetic anchovy	*Setipinna phasa*	Phassa	Whole fish	Pelagic
	Bigeye snapper	*Lutjanus lutjanus*	Lutjan	Whole fish	Benthopelagic
	Gangetic whiting	*Sillaginopsis panijus*	Tular Dandi	Fillet with skin	Demersal

### 2.2 Sample preparation

Before dissection, the total length and total weight of the experimental fishes were recorded. The average length and weight of six commercially important freshwater and marine water fishes are presented in Table 2. Following that, the fish was dissected using a sterilized stainless-steel knife. The edible portion, which consists of the muscle, skin, and bones, was sliced and crushed into small pieces to represent the parts that the people consumed. The detailed materials and methods for the proximate composition analysis of selected species are as follow.

**Table 2:** The average weight and length of commercially important six freshwater and six marine water fish species of Bangladesh.

Categories	Scientific Name	Mean ± SE	
		Length (cm)	Weight (g)
Freshwater fish	*Labeo rohita*	36.20 ± 1.36	657.91 ± 8.12
	*Oreochromis mossambicus*	25.40 ± 0.84	276.36 ± 3.18
	*Pangasius pangasius*	42.70 ± 1.82	616.83 ± 7.11
	*Clarias batrachus*	27.30 ± 1.03	183.52 ± 4.72
	*Polynemus paradiseus*	10.70 ± 0.62	26.78 ± 1.82
	*Channa striata*	31.80 ± 2.41	264.59 ± 7.53
Marine water fish	*Lepturacanthus savala*	30.60 ± 0.96	136.94 ± 4.62
	*Lates calcarifer*	42.30 ± 1.62	804.74 ± 9.53
	*Mugil cephalus*	23.70 ± 2.52	274.35 ± 5.83
	*Setipinna phasa*	17.90 ± 0.95	164.47 ± 3.68
	*Lutjanus lutjanus*	15.80 ± 1.47	104.83 ± 4.17
	*Sillaginopsis panijus*	20.70 ± 2.61	91.94 ± 6.36

### 2.3 Proximate composition analysis

The proximate composition of fish samples were analyzed in the Fish Nutrition Laboratory under the Department of Aquaculture at Bangladesh Agricultural University by following Association of Official Analytical Chemists (AOAC, 2000) methods with slight modifications.

#### 2.3.1 Crude protein (%) analysis

The protein content was calculated by transforming total nitrogen to crude protein using Kjeldahl's technique (Bloc Digest 12, JP Selecta, Spain) with slight modification (AOAC, 1990; Anderson and Ingram, 1993). In brief, known quantities of sample (approximately 0.5 g), catalyst mixture (1.1 g) and concentrated H_2_SO_4_ (10 ml) was taken in a Kjeldahl flask and was digested in digestion unit (Digestor, Model-2020) for 45 min to obtain a clear solution. The digest was then distilled in distillation unit (Kjeltec system, Distilling unit, Model-1026) using 33% sodium thiosulphate (Na_2_S_2_O_3_), 40% sodium hydroxide (NaOH) and 4% boric acid solution and was titrated with standard hydrochloric acid (HCl). The percentage (%) of total nitrogen was multiplied by the empirical factor of 6.25 assuming that protein contains 16% nitrogen.

The % Nitrogen was calculated by using the formulae, % Nitrogen = [Milliequivalent of nitrogen (0.014) × titrant value (ml) × Strength of HCl/Sample weight (g)] x 100. Then the crude protein (%) was calculated by multiplying % Nitrogen and conversion factor 6.25. Each sample have at least three replicates.

#### 2.3.2 Crude lipid estimation

The crude lipid content was determined by adopting the technique of Bligh and Dyer (1959) with slight modifications. Briefly, the weighed fish sample (extract sample) were kept in acetone for 6 h in a Soxhlet apparatus. The oil obtained was collected in a small pre-weighed beaker and kept in oven for 20 min to evaporate the acetone. Oil in the beaker was then weighed on an electronic balance and the percentage of total lipid was calculated using the formula, Total lipid (%) = [Weight of lipid (g) × weight of sample (g)] × 100. Each sample have at least three replicates.

#### 2.3.3 Ash content analysis

Ash content was determined by igniting the sample in muffle furnace (ELM 11/6 UK) at 550 °C for 20 h. The ash content (%) was determined by using the formulae, Ash content (%) = [Weight of ash (g) × weight of sample (g)] × 100. Each sample have at least three replicates.

#### 2.3.4 Moisture content (%) analysis

The moisture content (%) was determined in triplicate by placing an accurately weighed amount (about 2–3 g) ground sample in a pre-weighted porcelain crucible in a thermostat oven (HAS/50/TDIG/SS, Hot Air Oven, Genlab, UK) (AOAC, 1990) at 105 °C for 24 h until a constant weight was obtained. The loss of weight was calculated as percentage moisture content. The moisture content (%) was analysed by the following equation, Moisture content (%) = [{Original Sample Weight (g) – Dried Sample Weight (g)}/Original Sample Weight (g)] × 100.

#### 2.3.5 Total carbohydrate content (%) analysis

The total carbohydrate content (%) was calculated by subtracting the amount of protein, lipid, ash, and moisture from 100 (Onyeike et al., 2000). For each species, three replicates were used in the analyses. The equation is total carbohydrate content (%) = [100- (moisture + crude protein + crude lipid + ash + crude fibre)].

### 2.4 Mineral content analysis

The fish samples (tissue sample, Table 2) were chopped, air dried and kept in labeled brown paper packet. The samples were dried in an oven at 70 °C for 48 h in an electric oven (Heraeus, Germany) and for proximate analysis, the mixture was fully blended in a food blender with stainless steel blades. For mineral analysis, 0.5 g from each replicated fish sample was taken separately into a clean dry digestion flask. Ten mL of di-acid solution (HNO_3_:HClO_4_ in a 2:1 ratio) was then poured into it. After keeping overnight, the flask was heated with a digestion chamber (BUCHI Digest System, K-437, Switzerland) at a temperature gradually elevated to 180 ᴼC (Islam et al., 2019). The flask contents were heated until they were completely translucent. The digest was chilled and filtered using Whatman No. 42 filter paper after being diluted with distilled water. With distilled water, the volume was increased to 100 mL and stored in a sealed plastic container. All of the fish extracts were preserved at 4ᴼC until chemical analyses were done (Tandon, 1995). Analytical reagent (AR) grade chemicals were used in all cases.

Sodium (Na) and potassium (K) were determined using a flame emission spectrophotometer (Spectrolab, UK) with suitable filters. Moreover, calcium (Ca) and magnesium (Mg) were determined by the atomic absorption spectrophotometer (Model Varian, AAS Spectra 55B, Australia). Furthermore, phosphorus (P) and sulfur (S) was evaluated following appropriate color development with a double beam UV-VIS spectrophotometer (AOAC, 2000; Islam et al., 2019). Throughout the elemental analysis, each batch contained the appropriate metal standard, blank, triplicate, and ongoing calibration verification (AOAC, 2000).

### 2.5 Energy content

The Crisan and Sands' (1978) method was used to quantify the energy content of the six freshwater and six marine water fish species based on their crude protein, lipid, and carbohydrate content.

### 2.6 Statistical analysis

All data are presented as a mean ± standard error. Using the SPSS (Statistical Package for Social Science, version 25.0, SPSS Inc., Chicago, Illinois, USA) and R software, the statistical significance of the differences between means was determined using One-way ANOVA, followed by Tukey's multiple comparisons, and Kruskal-Wallis non-parametric tests. The principal component analysis (PCA) was performed using software R to determine the variation in the proximate composition and mineral contents among 12 fish species of freshware and marine water origin. The dimension results of the PCA bi-plots were used to demonstrate the affinity among these species. Significant differences were defined as values having a *p* < 0.05. The proportion data was transformed to normalize before statistical analysis.

## 3 Results and discussion

### 3.1 Proximate composition

The biochemical content of fish varies depending on the species. Table 3 presents the proximate composition of six commercially important freshwater and six commercially important marine water fish species in Bangladesh.

**Table 3:** Proximate composition (%) of commercially important six freshwater and six marine water fish species of Bangladesh.

Categories	Species	Protein (%)	Lipid (%)	Ash (%)	Moisture (%)	Carbohydrates (%)	Total energy (Kcal)
Freshwater fish	*L. rohita*	18.41 ± 0.15^a^	3.03 ± 0.11^a^	1.04 ± 0.03^a^	77.35 ± 0.20^a^	0.17 ± 0.09^a^	133.62 ± 4.74^a^
	*O. mossambicus*	18.73 ± 0.21^a^	1.91 ± 0.05^c^	1.62 ± 0.04^c^	77.61 ± 0.17^a^	0.13 ± 0.03^a^	124.74 ± 5.68^b^
	*P. pangasius*	15.62 ± 0.10^c^	3.70 ± 0.10^b^	0.85 ± 0.08^a^	79.11 ± 0.19^b^	0.72 ± 0.23^c^	126.22 ± 3.47^b^
	*C. batrachus*	16.33 ± 0.05^d^	1.25 ± 0.06^e^	1.12 ± 0.07^a^	81.16 ± 0.05^c^	0.15 ± 0.06^a^	104.98 ± 6.26^c^
	*P. paradiseus*	17.33 ± 0.27^b^	2.88 ± 0.16^a^	1.31 ± 0.29^b^	77.97 ± 0.19^a^	0.50 ± 0.10^b^	127.40 ± 6.25^b^
	*C. striata*	18.02 ± 0.16^a^	0.32 ± 0.03^d^	1.16 ± 0.03^a^	80.40 ± 0.15^bc^	0.10 ± 0.01^a^	105.71 ± 4.37^c^
Marine water fish	*L. savala*	18.83 ± 0.15^ae^	2.22 ± 0.11^c^	1.19 ± 0.07^a^	77.29 ± 0.27^a^	0.47 ± 0.21^bd^	129.63 ± 7.36^ab^
	*L. calcarifer*	18.67 ± 0.25^a^	5.04 ± 0.09^f^	4.35 ± 0.21^d^	71.75 ± 0.32^d^	0.19 ± 0.05^a^	153.97 ± 8.36^d^
	*M. cephalus*	16.73 ± 0.3^b^	13.40 ± 0.46^g^	1.17 ± 0.02^a^	68.34 ± 0.31^e^	0.36 ± 0.13^de^	221.77 ± 9.75^e^
	*S. phasa*	17.27 ± 0.08^b^	2.13 ± 0.10^c^	3.22 ± 0.08^e^	77.23 ± 0.13^a^	0.16 ± 0.06^a^	118.64 ± 6.45^b^
	*L. lutjanus*	18.77 ± 0.16^a^	2.68 ± 0.13^ac^	1.28 ± 0.24^a^	76.94 ± 0.20^af^	0.33 ± 0.11^e^	132.99 ± 5.67^a^
	*S. panijus*	17.42 ± 0.08^b^	4.03 ± 0.16^b^	1.61 ± 0.09^bc^	76.58 ± 0.12^f^	0.35 ± 0.06^de^	138.11 ± 7.48^af^

Different superscript in a row differs significantly (*p* < 0.05).

Protein content was found to differ significantly (*p* < 0.05) across the twelve species. Protein content in the freshwater fishes ranged from 15.616% (*Pangasius pangasius*) to 18.730% (*Oreochromis mossambicus*) but in the case of marine water fish, the protein content ranged from 16.733% (*Mugil cephalus*) to 18.826% (*Lepturacanthus savala*). Because fish is an animal-source protein, its total protein level ranged from 11.9 to 20.6 percent, implying that it is of good nutritional quality (WHO, 2007). The protein content in the marine water fishes was comparatively higher than the freshwater fishes. In addition, fish proteins are rich in essential amino acids, especially lysine, methionine, and taurine, which are insufficient in other sources of animal muscle, and some of the other amino acids such as glycine, alanine, aspartic acid, and glutamic acid that are responsible to produce the flavor and taste (Kim, 2020).

The lipid content in freshwater fishes ranges from 0.316% (*Channa striata*) to 3.696% (*Pangasius pangasius*) and in the case of marine water fish, the lipid content ranged from 2.130% (*Setipinna phasa*) to 13.396% (*Mugil cephalus*) (Table 3). The lipid content differs significantly among the fish species. The lipid content of marine fish is greater than that of freshwater fish. It is commonly believed that consuming Saturated fatty acids (SFAs) is linked to a higher risk of developing coronary heart disease and higher levels of LDL cholesterol (Santos et al., 2013). The fish lipid is a rich source of polyunsaturated fatty acids such as eicosapentaenoic acid (EPA) and docosahexaenoic acid (DHA), both of which have been shown to have benefits for the human improvement in several types of disease (Saoud et al., 2008; Panchal and Brown, 2021). Numerous studies on the Kuopio Ischaemic Heart Disease and the Nurses' Health Study have discovered an inverse relationship between the PUFA/SFA ratio and cardiovascular outcomes, implying that substituting PUFA for SFA in the diet will reduce cardiovascular disease (Fung et al., 2009).

The ash contents of the freshwater and marine water fishes also differ significantly. The highest ash content was found at 4.350% in the case of *Lates calcarifer* (marine water fish) and the lowest ash content was found at 0.850% in the case of *Pangasius pangasius* (freshwater fish) (Table 3). The ash (0.850–4.350%) levels were well within the range covered by most of the species (Badiani et al., 1996; Ullah et al., 2022).

The moisture content differs significantly among the twelve fish species. The moisture content found in freshwater fishes ranged from 77.346% (*Labeo rohita*) to 81.160% (*Clarias batrachus*), but moisture content in marine water fishes ranged from 68.343% (*Mugil cephalus*) to 77.293% (*Lepturacanthus savala*) (Table 3). These findings revealed that fish are mostly made up of moisture. The moisture content of fish has been estimated to be between 70% and 80% of the body weight (Ackman, 1989). Moisture content in freshwater fishes was relatively higher than in marine water fishes.

Carbohydrate content was very low (0.103%–0.723%) in the freshwater and marine water fish species but it differs significantly among the twelve fish species. This is characteristic of striated muscle, where glucose is found as glycogen and as a component of nucleotide chemical components (FAO, 2005). The quantity of glucose in white fish flesh is usually little in terms of dietary importance (Murray and Burt, 1969).

**Figure 1:**
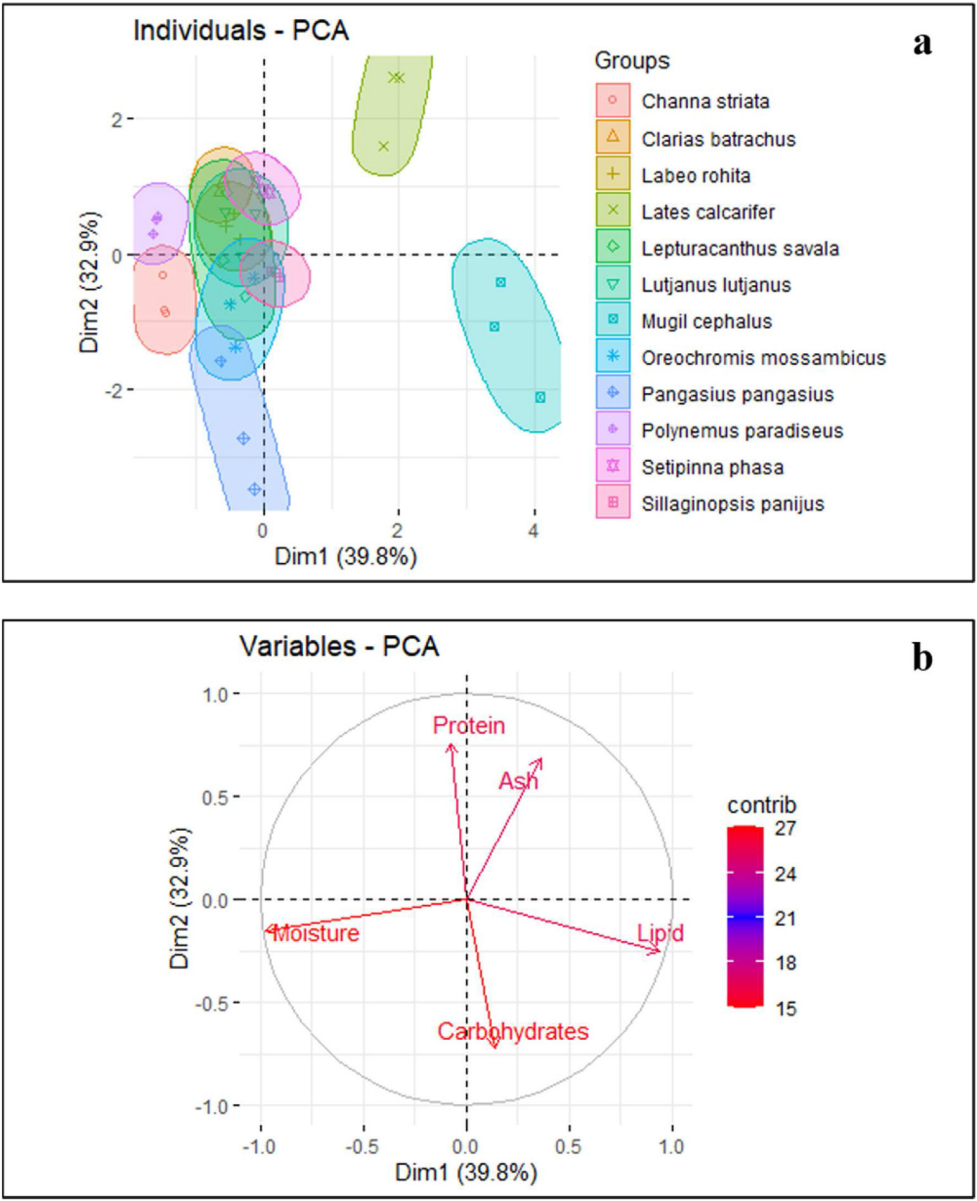
Six freshwater and six marine fishes were included in the PCA score and biplot, along with various measurable factors such as protein, lipid, carbohydrate, and ash. The graph showed positive and negative associations between these fish groups (a) bi-plots of PCA, and (b) dimension results denote the loadings of every character.

Among the six freshwater and six marine water fishes, only the *Lates calcarifer* and *Mugil cephalus* were grouped together in the PCA biplot's positive site with ash, lipid, and carbohydrate (Figures 1a, 1b). However, protein and moisture enabled these two fish species to be distinguished from the other 10 fish species that had gathered in the PC2 negative zone. Additionally, the first principal component (PC1) accounted for 39.8% of the difference, followed by the second principal component (PC2) at 32.9%, the third principal component (PC3) at 14.03%, and the fourth principal component (PC4) at 13.24% and the fifth principal component (PC5) at 0.03%. The bi-plot showed that the values for 10 species, including 6 freshwater fish and 4 marine fish, were intermingled and overlapped, while *Lates calc*arifer and *Mugil cephalus* formed distinct groups that were positively associated.

Proximate composition varies between freshwater and marine water fishes. The protein, lipid, and ash content were significantly higher in marine water fishes than in freshwater fishes. Food, gender, age, habitat, and weather all have an impact on chemical composition, which varies widely between species and individuals (Obodai et al., 2009; Ondo-Azi et al., 2013; Osibona, 2011). The results of the study revealed that the energy content of the six freshwater and six marine water fish species varies significantly (Table 3). The energy content of marine water fish species was substantially higher than that of freshwater fish species. The nutritional state, growth, and stress variables that affect the fish are all closely related to the energy content (Roy et al., 2020). Energy deficiency syndrome was caused by a lack of energy in fish, which resulted in severe physiological abnormalities, illnesses, and mortality (Murray and Burt, 1969; Obodai et al., 2009). The energy state of fish samples and their nutrients must be examined in a variety of contexts, including ichthyological, ichthyo-pathological, and ecological studies (Schreckenbach and Spangenberg, 1987). The technique for measuring the gross energy content in fishes by their dry matter content, as described in this work, is a straightforward and effective way of calculating freshwater and marine water fishes in natural water bodies, gravel pits, ponds, and aquaculture facilities.

### 3.2 Mineral content

Mineral content differs significantly among the six freshwater and six marine water fish species (Table 4). The calcium content range in freshwater fish species were between 0.555% (*Pangasius pangasius*) and 1.671% (*Polynemus paradiseus*) in wet sample while calcium content in marine fishes was between 0.676% (*Lates calcarifer*) and 3.495% (*Setipinna phasa*). The calcium concentration of freshwater and marine fish samples was significantly different. Irwandi and Farida (2009) found calcium concentrations ranging from 0.57% to 3.03% in the samples which are more or less similar to our current findings. The mean concentrations of magnesium ranged between 0.281% (*Pangasius pangasius*) and 1.027% (*Polynemus paradiseus*) in freshwater fishes but in marine water fishes, it ranged from 0.544% (*Sillaginopsis panijus*) to 1.885% (*Setipinna phasa*). The investigation of Erkan and Ozden (2007) is comparable to the current study. Magnesium levels in freshwater fish were substantially lower than those in marine fish. Changes in magnesium concentration in our studies might be attributable to differences in species, capture area, and a variety of other physical and environmental factors.

**Table 4:** Mineral contents (%) of commercially important six freshwater and six marine water fish species of Bangladesh.

Categories	Species	Ca (%)	Mg (%)	P (%)	Na (%)	K (%)	S (%)	Na/K	Ca/P
Freshwater fish	*L. rohita*	1.31 ± 0.01^a^	0.39 ± 0.02^a^	1.12 ± 0.05^a^	0.33 ± 0.02^a^	1.28 ± 0.04^a^	0.48 ± 0.04^a^	0.26 ± 0.03^a^	1.17 ± 0.05^a^
	*O. mossambicus*	1.62 ± 0.02^b^	0.41 ± 0.01^ac^	1.21 ± 0.06^a^	0.42 ± 0.06^a^	1.13 ± 0.07^ab^	0.68 ± 0.06^ab^	0.37 ± 0.04^ab^	1.36 ± 0.04^b^
	*P. pangasius*	0.56 ± 0.02^c^	0.28 ± 0.03^a^	0.91 ± 0.06^b^	0.42 ± 0.05^a^	1.06 ± 0.05^ab^	0.92 ± 0.08^c^	0.39 ± 0.03^b^	0.61 ± 0.03^c^
	*C. batrachus*	0.74 ± 0.01^c^	0.41 ± 0.01^ac^	0.95 ± 0.04^b^	0.39 ± 0.05^a^	1.26 ± 0.06^ab^	0.82 ± 0.09^bc^	0.31 ± 0.03^ab^	0.78 ± 0.05^e^
	*P. paradiseus*	1.67 ± 0.03^b^	1.03 ± 0.07^b^	1.21 ± 0.03^a^	0.38 ± 0.03^a^	0.86 ± 0.05^b^	0.61 ± 0.03^ab^	0.44 ± 0.01^b^	1.38 ± 0.07^b^
	*C. striata*	0.84 ± 0.02^c^	0.37 ± 0.04^a^	0.93 ± 0.02^b^	0.34 ± 0.05^a^	1.43 ± 0.09^c^	0.79 ± 0.05^bc^	0.24 ± 0.06^a^	0.90 ± 0.01^d^
Marine water fish	*L. savala*	0.71 ± 0.02^c^	0.65 ± 0.08^cd^	0.83 ± 0.05^b^	0.72 ± 0.03^b^	0.91 ± 0.03^a^	0.55 ± 0.03^a^	0.79 ± 0.08^c^	0.86 ± 0.01^e^
	*L. calcarifer*	0.68 ± 0.01^c^	0.61 ± 0.02^cd^	0.91 ± 0.03^b^	0.45 ± 0.02^a^	1.46 ± 0.16^c^	1.25 ± 0.06^d^	0.31 ± 0.06^ab^	0.75 ± 0.04^ef^
	*M. cephalus*	2.58 ± 0.11^d^	1.65 ± 0.09^e^	1.75 ± 0.08^c^	0.78 ± 0.04^b^	1.12 ± 0.01^ab^	1.33 ± 0.06^d^	0.70 ± 0.04^c^	1.48 ± 0.16^b^
	*S. phasa*	3.49 ± 0.23^e^	1.89 ± 0.13^f^	2.11 ± 0.02^d^	0.45 ± 0.04^a^	1.09 ± 0.06^ab^	0.87 ± 0.04^c^	0.42 ± 0.07^bd^	1.65 ± 0.15^g^
	*L. lutjanus*	2.57 ± 0.13^d^	1.37 ± 0.04^g^	1.56 ± 0.03^e^	0.48 ± 0.04^a^	0.95 ± 0.03^ab^	0.81 ± 0.08^c^	0.50 ± 0.03^e^	1.65 ± 0.06^g^
	*S. panijus*	1.29 ± 0.04^a^	0.54 ± 0.02^ac^	0.85 ± 0.03^b^	1.68 ± 0.08^c^	0.85 ± 0.04^b^	0.56 ± 0.06^ab^	1.99 ± 0.24^f^	1.53 ± 0.11^bg^

Different superscript in a row differs significantly (*p* < 0.05).

The phosphorus content in freshwater fishes ranged from 0.906% (*Pangasius pangasius*) to 1.213% (*Polynemus paradiseus*) while the phosphorus content in marine water fish samples ranges between 0.826% (*Lepturacanthus savala*) and 2.114% (*Setipinna phasa*). The phosphorus content differs significantly among the twelve freshwater and marine water fishes but it was comparatively higher in the marine water fishes. Gokoglu et al. (2004) found phosphorous content of 3.37% which was higher compared to the current study. All of the samples had quite low sodium contents. The sodium contents in freshwater fish samples ranged between 0.332% (*Labeo rohita*) to 0.419% (*Pangasius pangasius*) and in marine water fish samples, it ranged from 0.450% (*Lates calcarifer*) to 1.681% (*Sillaginopsis panijus*). Lower Na and greater K concentrations were found, making our fish an excellent meal for public health, particularly in the prevention of cardiovascular disease (Perez et al., 2014; Bu et al., 2012). For freshwater fish, the lowest and highest content of potassium was recorded in *Polynemus paradiseus* (0.858%) and *Channa striata* (1.429%), respectively. For marine fish, *Sillaginopsis panijus* had the lowest value (0.846%), while *Lates calcarifer* had the highest value (1.458%). In comparison to the current findings, Erkan and Ozden (2007) found much greater potassium levels, averaging 3.93–4.59 g/100g. The sulfur content in freshwater fish species varied between 0.480% (*Labeo rohita*) and 0.923% (*Pangasius pangasius*) while in the marine water fish samples it ranged between 0.549% (*Lepturacanthus savala*) and 1.326% (*Mugil cephalus*). Minerals are are essential building blocks for many enzymes and metabolic processes, as well as contributing to fish growth. Only a little amount of these minerals are found in the human body, and a deficiency in these critical nutrients causes a plethora of issues, including lower productivity and disease (Marichamy et al., 2012). Except for *Sillaginopsis panijus*, all of the fish samples had a Na/K value of less than 1. Lower quantities of Na and larger concentrations of K were discovered, transforming our fish into a healthy meal for humans, particularly in the prevention of heart disease. The ratio of sodium to potassium in our meals should be lower than 1 unless we are aiming to prevent cardiovascular disease (Osibona, 2011; Bu et al., 2012; Valenzuela et al., 2020). The Ca/P ratio yielded a range of results. It was found in roughly 1% of all freshwater and marine fish samples. The primary structural minerals, Ca and P, are found in equal amounts in the mineral bone matrix (Liu et al., 2008; Bu et al., 2012; Toppe et al., 2007). The ratio of calcium to phosphorus in meals should be around 1 (Belitz et al., 2009).

**Figure 2:**
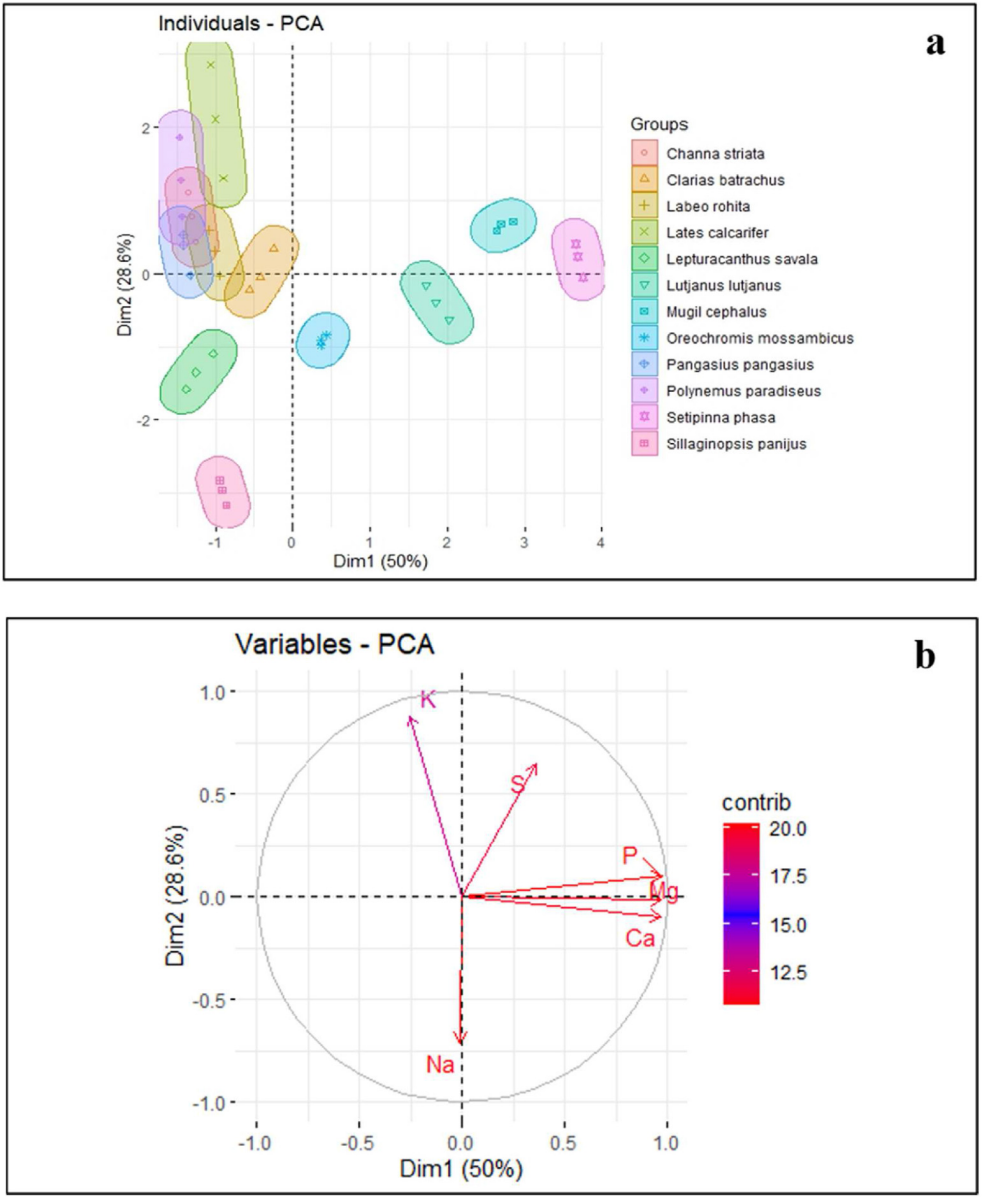
Six freshwater and six marine fishes were included in the PCA score and biplot, along with various measurable factors such as sodium (Na), potassium (K), sulfur (S), phosphorus (P), magnesium (Mg), and calcium (Ca). The graph showed positive and negative associations between these fish groups (a) bi-plots of PCA, and (b) dimension results denote the loadings of every character for minerals.

Among the studied 12 fish species of freshwater and marine water origin, *Setipinna phasa*, *Mugil cephalus*, *Lutjanus lutjanus*, and *Oreochromis mossambicus* were found to be grouped together in the PCA biplot's positive site with magnesium, phosphorus, calcium, and sulfur (Figures 2a, 2b). However, sodium and potassium made it possible to identify the remaining eight fish species that had gathered in the PC2 negative zone. Additionally, the first principal component (PC1) was responsible for 50% of the difference, with the second principal component (PC2) coming in at 28.6%, the third principal component (PC3) at 14.52%, the fourth principal component (PC4) at 5.15%, the fifth principal component (PC5) at 1.32%, and the sixth principal component (PC6) at 0.41%. The bi-plot revealed that values for 8 species, including 5 freshwater fish and 3 marine fish, overlapped and mixed together, however that *Setipinna phasa*, *Mugil cephalus*, *Lutjanus lutjanus*, and *Oreochromis mossambicus* formed unique groups that were positively linked.

### 3.3 Price comparison

The price range of freshwater fishes ranged from 2.25-10.50 USD/Kg where the lowest price in *Oreochromis mossambicus* and the highest price in *Pangasius pangasius* (Table 5). But in the case of marine water fishes, the price ranged from 1.25-6.25 USD/Kg where the lowest price in *Setipinna phasa* and *Lutjanus lutjanus* and the highest price in *Lates calcarifer* (Table 5). The price of freshwater fishes was comparatively higher than marine water fishes.

**Table 5:** Price (USD/Kg) of commercially important six freshwater and six marine water fish species of Bangladesh.

Categories	Scientific name	Price (USD/Kg)
Freshwater fish	*Labeo rohita*	3.75–4.25
	*Oreochromis mossambicus*	2.25–2.75
	*Pangasius pangasius*	10.00–10.50
	*Clarias batrachus*	8.75–10.00
	*Polynemus paradiseus*	6.75–7.50
	*Channa striata*	7.50–8.75
Marine water fish	*Lepturacanthus savala*	3.00–3.75
	*Lates calcarifer*	5.00–6.25
	*Mugil cephalus*	4.25–5.00
	*Setipinna phasa*	1.25–1.75
	*Lutjanus lutjanus*	1.25–1.75
	*Sillaginopsis panijus*	2.50–3.00

The marine water fishes were a good source of the different proximate composition except for the moisture content than freshwater fishes. In addition, the different mineral contents were also significantly higher in the marine water fishes except for potassium content. Energy content was also higher in marine water fishes than in freshwater fishes. Moreover, the price of marine water fishes was also lower than the freshwater fishes. As the marine water fishes were an easily available and good source of different proximate and mineral content, the marine fishes can be a good food item to the people of our country in terms of economy, nutrition and could provide better health benefits.

## 4 Conclusion

The comparative information on the proximate and mineral content of freshwater and marine fish species of the coast of Bangladesh was lacking. So, this research looked at the nutritional characteristics of both freshwater and marine water fish species. The findings showed that the tested species of fish are decent sources of a variety of essential nutrients and minerals, with marine fishes having the highest concentration. Fish from the sea are abundant in protein and include a wide range of minerals. These species are both safe to eat and beneficial to one's health. Although this result offers useful evidence on the nutrition of some of Bangladesh's most widely consumed fish species, more intensive research is required on essential and non-essential amino acids, and fatty acids (saturated and unsaturated) to produce composition standards.

## Declarations

### Author contribution statement

Md. Rahamat Ullah: Performed the experiments; Wrote the paper.

Md. Arifur Rahman: Contributed reagents, materials, analysis tools or data; Wrote the paper.

Md. Nazmul Haque: Analyzed and interpreted the data.

Md. Rajib Sarkar: Conceived and designed the experiments, Analyzed and interpreted the data.

M. Muhsinul Islam: Performed the experiments.

Md. Ariful Alam: Conceived and designed the experiments.

### Funding statement

Dr. Md. Ariful Alam was supported by Research and Training Center (RTC), 10.13039/501100014587Patuakhali Science and Technology University [4829 and 5921 (Fish-07 and Fish-01)].

### Data availability statement

Data will be made available on request.

### Declaration of interest's statement

The authors declare no conflict of interest.

### Additional information

No additional information is available for this paper.
